# Decreased miR-329-3p upregulates *Adamts4* and *Dnajb1* in mouse hepatic I/R injury in an age-independent manner

**DOI:** 10.7150/ijms.87174

**Published:** 2023-09-18

**Authors:** Lin Zhu, Wu Duan, Bo Yang, Lan Wang

**Affiliations:** 1 Department of Pediatrics, Tongji Hospital, Tongji Medical College, Huazhong University of Science and Technology, Wuhan 430030, China.; 2 Division of Endocrinology, Department of Internal Medicine, Qilu Hospital of Shandong University, Jinan 250000, China.; 3 Department of Endocrinology, Tongji Hospital, Tongji Medical College, Huazhong University of Science and Technology, Wuhan 430030, China.; 4 Institute of Organ Transplantation, Tongji Hospital, Tongji Medical College, Huazhong University of Science and Technology, Wuhan 430030, China.; 5 Key Laboratory of Organ Transplantation, Ministry of Education, Ministry of Public Health, Chinese Academy of Medical Sciences, Wuhan 430030, China.; 6 Reproductive Medicine Center, Tongji Hospital, Tongji Medicine College, Huazhong University of Science and Technology, Wuhan 430030, China.

**Keywords:** Ischemia/reperfusion injury, liver surgery, microRNA, bioinformatics analysis, age-independent manner.

## Abstract

**Introduction**: Hepatic ischemia/reperfusion (I/R) injury is common after liver surgery, particularly in patients of older age. However, an understanding of the mechanism of injury remains incomplete. In this study, we explored the molecular mechanisms underlying hepatic I/R injury and associations with age in a murine model.

**Methods**: Gene expression profiling datasets (GSE72315 and GSE10654) and a microRNA (miRNA) expression profiling dataset (GSE72315) were downloaded from Gene Expression Omnibus. Differentially expressed genes (DEGs) and miRNAs (DEMiRs) were identified using online GEO2R or R before and after hepatic I/R injury in mice. Significant Gene Ontology (GO) terms were analyzed with the DAVID functional annotation tool. The DEMiR-miRNA target gene (miRTG) networks were constructed with miRTarBase, and the differentially expressed miRNAs and genes were analyzed with real-time quantitative polymerase chain reaction and immunofluorescence staining.

**Results**: Through bioinformatic analysis, seven novel candidate miRNAs were identified that may regulate the expression of nine genes in hepatic I/R injury. Before and after hepatic I/R injury, mmu-miR-9-5p, mmu-miR-329-3p, and mmu-miR-290a-5p showed significant differential expression both in young (1 month old) and old (1 year old) mice. miR-329-3p had the most significant differential expression, and its predicted target genes *Adamts4* and *Dnajb1* were also significantly upregulated.

**Conclusions**: The decrease in miR-329-3p expression upregulated *Adamts4* and *Dnajb1* expression in mouse hepatic I/R injury in an age-independent manner. This finding contributes to our understanding of hepatic I/R injury, and highlights novel molecular targets for future therapeutic development.

## Introduction

Hepatic ischemia/reperfusion (I/R) injury is a common pathophysiological process after liver surgery. It significantly affects both recovery of liver function and perioperative outcomes after liver resection and transplantation 1, 2. Hepatic I/R injury increases the risk of organ failure, acute and chronic tissue rejection, and satellite organ damage after liver transplantation; this limits the indications for liver resection and transplantation of marginal donor livers 3, 4. An aging global population has led to a corresponding increase in the age of both patients undergoing liver surgery and transplant donors. The compensatory capacity of the liver after I/R injury is significantly reduced with age, as such the rate of graft failure, postoperative complications, and mortality is increasing despite continuous improvements in perioperative and critical care 5, 6. Multiple alterations at the cellular and molecular levels contributed to the increased liver injury post‐IR in the aged mice. But to date, the relationship between donor age and sensitivity to I/R injury after liver transplantation and the effect of the former on the pathophysiological mechanism of the latter has not been fully elucidated.

MicroRNAs (miRNAs) are a class of small non-coding RNA molecules with a length of 22 nucleotides. It is considered a post-transcriptional regulator of gene expression and can bind to the 3 '-untranslated region (3' -UTR) of target messenger RNA (mRNA)^7^. More and more studies have shown that miRNAs are often implicated in the pathophysiology of liver disease and is thought to be involved in the development of toxic liver damage, viral hepatitis, and liver tumors 8-10. A number of miRNAs have been found to play a crucial role in liver I/R injury, such as miR-125b, miR-155, miR-182-5p, and so on^11^, ^12^. In this study, we amid to explore the differential expression of miRNAs and target genes between older and younger livers in a murine model of hepatic I/R injury using bioinformatic analysis. This sought to improve the understanding of the association between age and I/R injury, its underlying mechanism, and inform potential future therapeutic targets.

## Materials and Methods

### Gene Expression Omnibus (GEO) database analysis

The gene expression profiling datasets (GSE72315 and GSE10654) and a miRNA expression profiling dataset (GSE72315) were obtained from GEO (https://www.ncbi.nlm.nih.gov/geo/) of the National Center for Biotechnology Information (NCBI). Data from each microarray were separately analyzed with GEO2R or R (version 3.6.1) to identify DEGs and DEMiRs. Firstly, we explored the differential expression of miRNA and target genes in mice with and without I/R injury. Six mice with I/R and six sham-operated were adopted in GSE72315 (GPL13112, Illumina HiSeq 2000 platform) to profile miRNA expression. For the construction of the gene expression microarray, six mice with I/R and six sham-operated mice were included in an analysis using GSE10654 (GPL5759, Affymetrix GeneChip Mouse Genome 430 2.0 Array) and GSE72315 (GPL13112, Illumina HiSeq 2000 platform). The GSE10654 dataset was divided into two groups based on the mouse's age (1-month-old mice ('younger mice') versus 1-year-old mice ('older mice')). For miRNA expression profiling, the threshold was determined according to the following values: *P* < 0.05 and fold change ≥ 3.0. For each gene expression profiling, the threshold was determined according to the following values: *P* < 0.05 and fold change ≥ 1.5. We identified differentially expressed genes (DEGs) between the GSE72315 and GSE10654 datasets, and any differentially expressed miRNAs (DEMiRs) were grouped according to whether they were seen to be upregulated or downregulated^13^.

### Identification of miRNA target genes (miRTGs) and potential binding sites

We used miRTarBase to identify experimentally validated miRTGs. miRNA target DEGs (miRT-DEGs) were subsequently obtained from overlapping DEGs and miRTGs. Finally, high DEMiR-low miRT-DEGs and low DEMiR-high miRT-DEGs were derived from overlapping DEMiRs and miRT-DEGs. To outline the potential functions of these 31 upregulated and 58 downregulated DEGs, a functional enrichment analysis was performed. Significant GO terms were identified with the DAVID functional annotation tool.

### Animals

C57BL/6 male mice (1 month or 1 year) were purchased from the Beijing HFK Bio-Technology Company (Beijing, PR China). All experimental procedures involving animals were approved by the Ethics Committee of Tongji Hospital, Tongji Medical College, Huazhong University of Science and Technology. The mice received humane care according to the Guide for the Care and Use of Laboratory Animals of the Chinese Academy of Sciences. All animals were housed under standard environmental conditions with controlled temperature (22 ± 2°C), humidity (50 ± 10%), and light (12 h light/dark cycle) with free access to standard diet and water.

### Hepatic I/R injury mode

A hepatic I/R injury murine model was generated using a 70% hepatic I/R model 14. Induction of anesthesia was performed with an intraperitoneal injection of sodium pentobarbital (60 mg/kg), and all efforts were made to minimize suffering. The artery and portal vein were interrupted with an atraumatic clip for 60 min to block blood flow to the left and middle lobes of the liver. As the right liver lobe was excluded, mesenteric venous congestion was prevented by allowing portal decompression through the right and caudate liver lobes. During the operation, normothermia was maintained (37°C) using a warming pad and incubator. Maintenance of anesthesia was performed with intraperitoneally administered ketamine (100 mg/kg) and xylazine (20 mg/kg). Care was taken to avoid exsanguination from the abdominal aorta, and every effort was made to minimize any suffering of the animals throughout the study. Plasma samples and liver tissues were collected 6 hours after the liver I/R injury. Sham operation group (sham group): The remaining steps were the same as those I/R group except for blocking hepatic blood flow.

### Serum aminotransferase activities

To estimate the degree of hepatocyte damage after I/R injury, serum levels of alanine aminotransferase (ALT) and aspartate aminotransferase (AST) were measured using a Hitachi 7600 automatic analyzer (Hitachi, Ltd., Tokyo, Japan).

### Histopathological examination

Liver tissue samples were fixed with 10% formalin for 24 hours before being embedded in paraffin. The sections were stained with hematoxylin and eosin (H&E). Suzuki's histological grading was performed to evaluate for damage. This assessment has three components including sinusoidal congestion, hepatocyte necrosis, and ballooning degeneration. Each criterion was scored on a scale of 0 (no damage) to 4 (severe damage) depending on the degree of liver damage. In the absence of any congestion, vacuolization, or necrosis, the best score of 0 was assigned. Severe congestion accompanied by severe vacuolization and > 60% necrosis area was assigned a score up to 12.

### Terminal deoxynucleotidyl transferase dUTP nick end labeling (TUNEL) assay

Cell death in liver paraffin sections was detected using the TUNEL assay. The tissue samples were fixed in 10% neutral-buffered formalin before being embedded in paraffin. Hepatocyte death assays were performed with an *in-situ* cell death detection kit (Dead End Fluorometric TUNEL System; Promega, Madison, WI, USA) according to the manufacturer's instructions. The number of TUNEL-positive nuclei was counted in six randomly chosen images from non-overlapping areas of each group. Data were presented as the percentage of TUNEL-positive cells.

### RNA extraction and real-time quantitative polymerase chain reaction (RT-qPCR)

Total RNA, including miRNAs, was extracted from the liver tissue using TRIzol reagent (Invitrogen, USA). RNA concentration was assessed with NanoDrop 2000 (Wilmington, DE) and the levels of miRNAs were measured with qRT-PCR using Bulge-Loop™ miRNA qRT-PCR Starter Kit (RiboBio, Guangzhou, China) on ABI 7500 System (Applied Biosystems). The primers for miR-9-5p, miR-592-5p, miR-124-3p, miR-329-3p, miR-290a-5p, miR-709, miR-760-3p, and U6 small nuclear RNA were obtained from RiboBio Company (Guangzhou, China). The sequences are covered by a patent. miRNA expression levels were normalized to the expression of the internal control U6 using the 2^-ΔΔCT^ method 15.

### Immunofluorescence staining

Prepared paraffin sections from liver tissues were de-waxed in xylene twice for 15 minutes, dehydrated in graded concentrations of ethanol (5 minutes each), and then rinsed 3 times with 0.01 M phosphate-buffered saline (PBS; pH 7.4) (5 minutes each). The tissue sections on glass slides were placed in a humid box. Non-specific antibody binding was blocked by incubation with 10% bovine serum albumin for 30 min at 37°C. Primary antibodies to Adamts4 (diluted at 1:80) and Dnajb1 (diluted at 1:100) were added to the tissue sections and the sections were incubated in the humid box at 4°C overnight. After rinsing with PBS (pH 7.4) (3 times, 5 minutes each), the samples were incubated with fluorescent-tagged secondary antibodies (diluted at 1:100) in the dark, in the humid box at 37°C for 1 hour. Tissue sections were mounted in buffered glycerol, viewed under a fluorescence microscope, and photographed.

### Statistical analysis

All data are represented as mean ± standard error of the mean (SEM). Individual group statistical comparisons were analyzed by unpaired Student t-test, and the difference in mRNA expression between mice with hepatic I/R injury and controls was statistically analyzed by one-way analysis of variance using SPSS (Version 22.0). A value of *P* < 0.05 was considered statistically significant.

## Results

### Identification of aberrantly expressed miRNA-gene network in I/R

**Figure 1:**
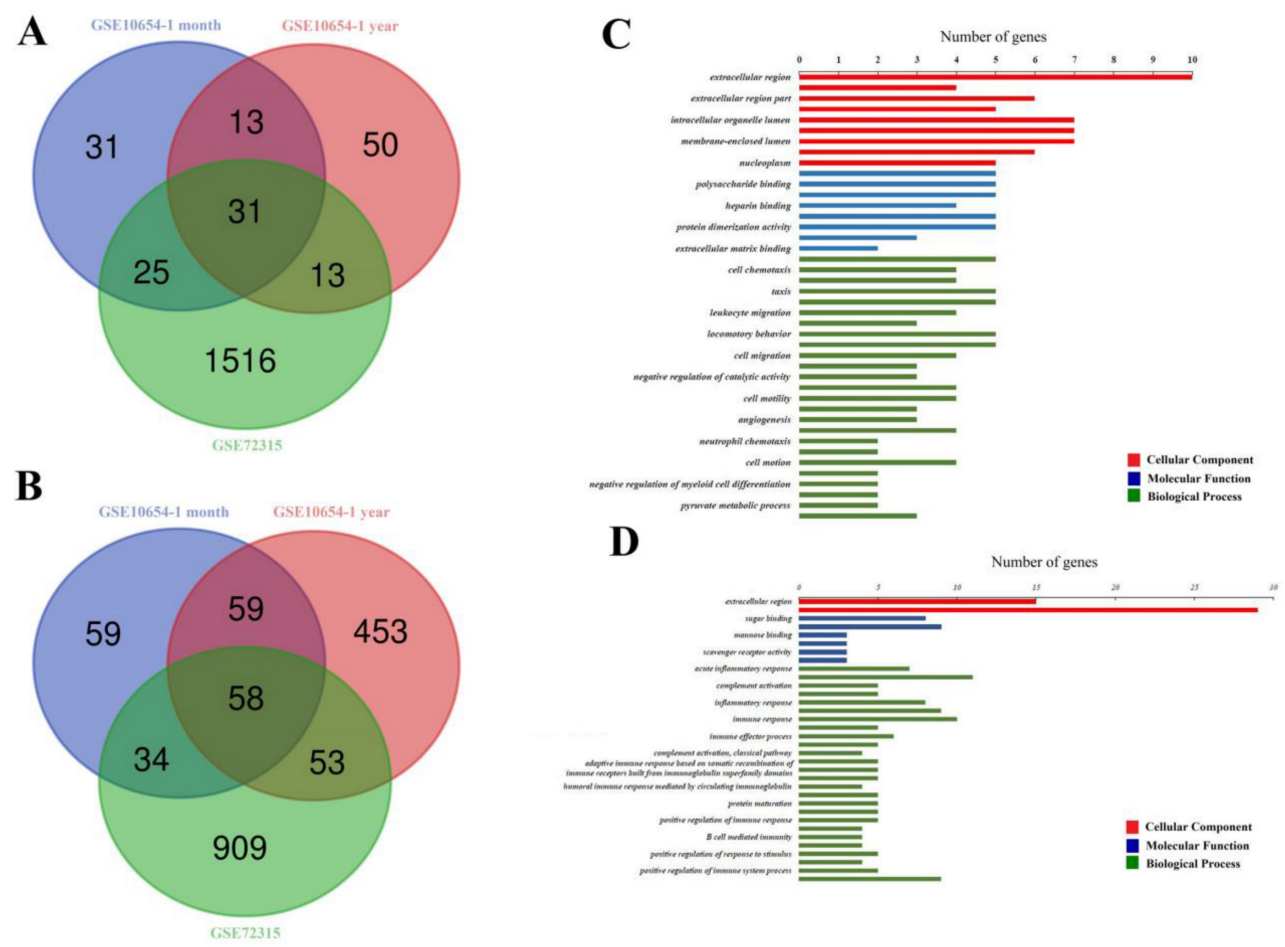
** Comparison of profiles of gene expression in following hepatic I/R injury. (A and B)** are Venn diagrams displaying the number of overexpressed** (A)** or downregulated **(B)** DEGs from datasets GSE72315 and GSE10654. **(C and D)** display significant GO terms. The cellular Component (CC) is displayed in red, the Molecular Function (MF) in blue, and the Biological Process (BP) in green. *P* < 0.05 (acquired from DAVID function annotation tool).

Thirty-one overlapping upregulated DEGs (100 in GSE10654-1-month-old subgroup, 107 in GSE10654-1-year-old subgroup, and 1585 in GSE72315, Fig. 1A) and 58 overlapping downregulated DEGs (210 in GSE10654-1-month-old subgroup, 623 in GSE10654-1-year-old subgroup, 1054 in GSE72315, Fig. 1B) were identified. Significant GO terms were exhibited in Figure 1C and D.

**Figure 2:**
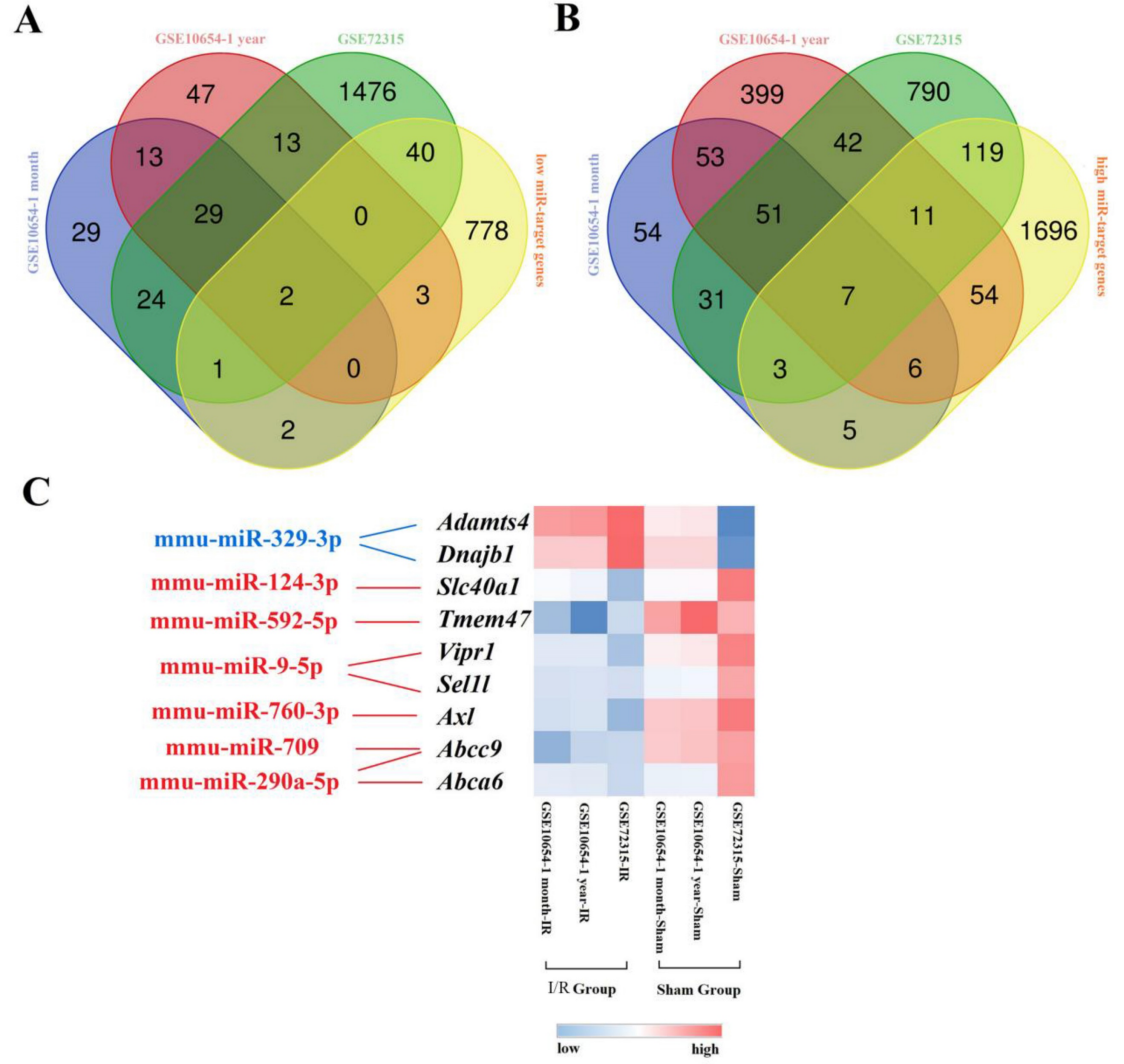
** Graphical representation of the aberrantly expressed miRNA-gene network following hepatic I/R injury. (A and B)** display Venn diagrams with the number of low DEMiR-high miRT-DEGs **(A)** and high DEMiR-low miRT-DEGs **(B)** following I/R injury from overlapping DEMiRs and miRT-DEGs. **(C)** is a heatmap of miRT-DEGs constructed based on the average linkage method and the Person's distance measurement method. Genes with higher expression are shown in red, while those with lower expression are shown in blue. Downregulated miRNAs are shown in blue and upregulated miRNAs are shown in red.

To identify aberrantly expressed miRNA-gene networks following I/R injury, GSE72315 was used for DEMiR screening. Sixty upregulated and 26 downregulated DEMiRs were detected. We used miRTarBase to subsequently identify 2688 upregulated and 1539 downregulated miRT-DEGs (Supplementary Materials). Finally, seven low DEMiR-high miRT-DEGs and two high DEMiR-low miRT-DEGs were obtained from overlapping DEMiRs and miRT-DEGs (Fig. 2A, B). In total, seven novel miRNAs, including mmu-miR-329-3p, mmu-miR-124-3p, mmu-miR-592-5p, mmu-miR-9-5p, mmu-miR-760-3p, mmu-miR-709, and mmu-miR-290a-5p, were thought to possibly regulate the expression of nine genes, including *Adamts4*, *Dnajb1*, *Slc40a1*, *Tmem47*, *Vipr1*, *Sel1l*, *Axl*, *Abcc9*, and *Abca6* (Fig. 2C).

### Association between age and severity of hepatic I/R injury in mice

**Figure 3:**
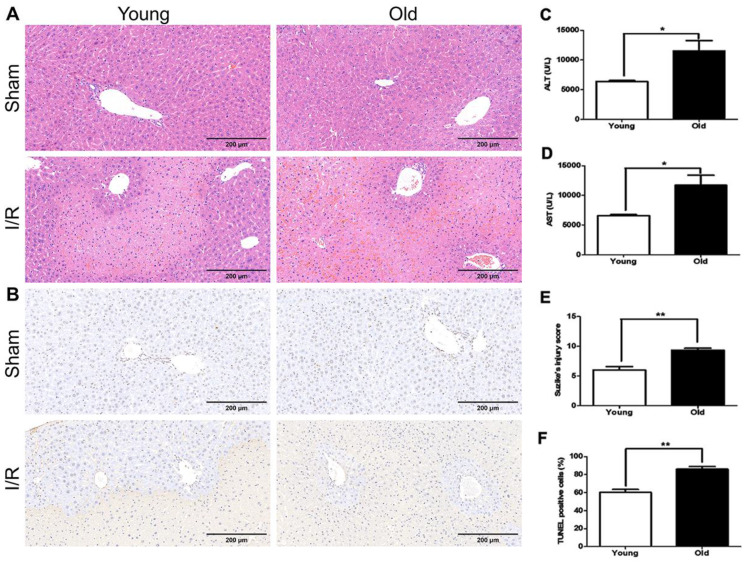
** The association between age and severity of hepatic I/R injury in mice.** Younger and older mice were subjected to hepatic I/R injury (I/R) or a sham operation (Sham). Liver and blood samples were collected 6 hours after reperfusion. **(A)** Representative histology of the liver as analysed with H&E staining. **(B)** Representative images of TUNEL staining of liver tissues. **(C/D)** The serum levels of ALT and AST as measured after reperfusion. **(E)** The pathological score of liver damage after hepatic I/R injury.** (F)** The quantification of TUNEL-positive cells in liver tissues. The data are expressed as mean ± SEM (*P < 0.05, **P < 0.01, young I/R versus old I/R group).

Histopathological examination revealed that the 'older mice' group had much more significant morphological alterations in the liver at 60 min after reperfusion than in younger mice, including severe damage to the morphology of the liver lobe, excessive hemorrhage and necrosis, swollen hepatocytes, and inflammatory cell infiltration (Fig. 3A, E). The number of TUNEL-positive cells increased more in older mice than in younger mice (Fig. 3B, F). In addition, the serum levels of ALT and AST were higher in older mice than in younger mice (Fig. 3C, D). Together these data suggest more significant hepatic I/R injury in older than younger mice in this model.

### Differential expression of miRNAs and target genes before and after hepatic I/R injury in young and older mice

**Figure 4:**
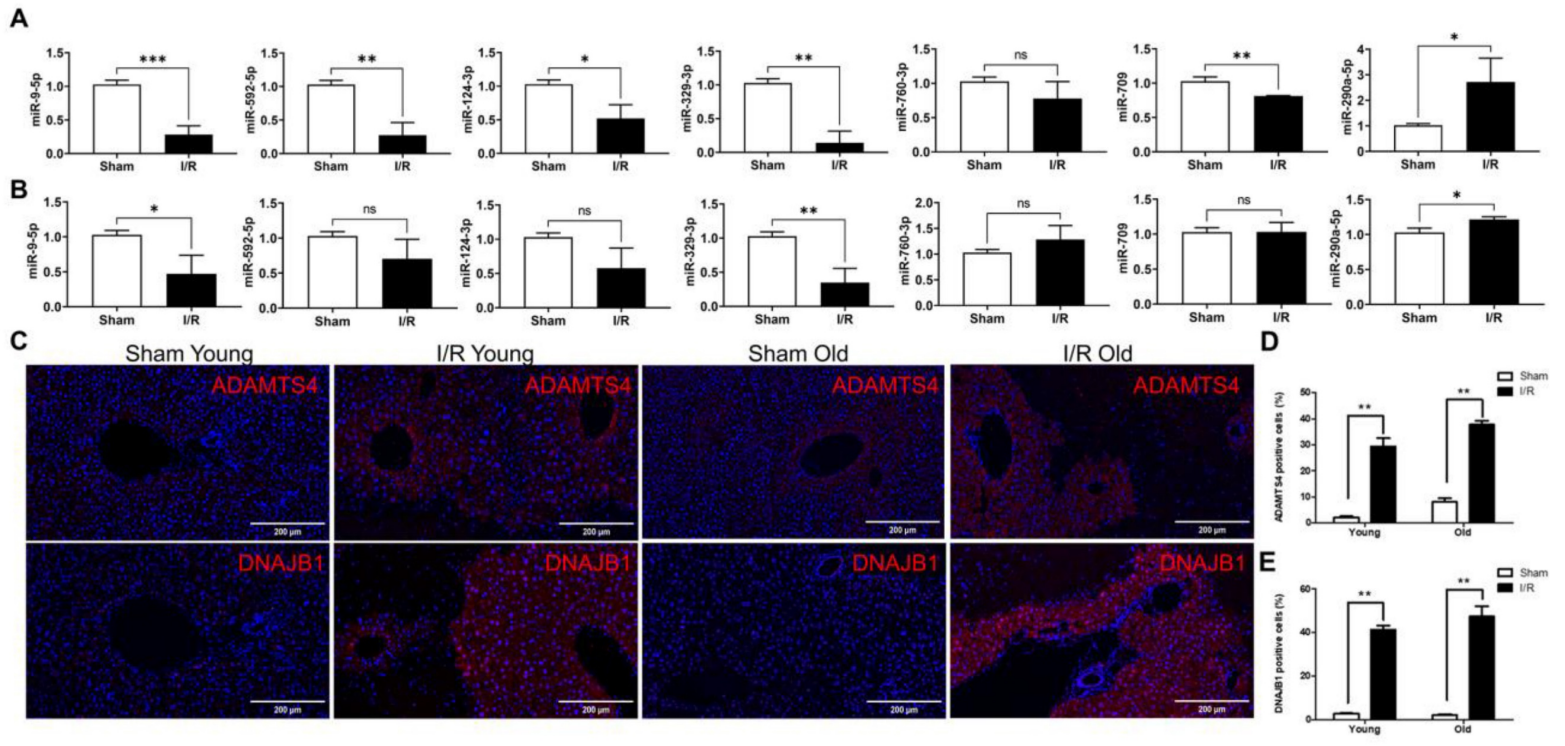
** The differential expression of miRNAs and selected target genes after hepatic I/R injury in younger versus older mice. (A and B)** show the seven differentially expressed miRNAs after hepatic I/R injury in younger **(A)** and older mice **(B)** using qRT-PCR. miRNA expression levels were normalized to the expression of the internal control U6. **(C)** Liver tissues were stained with specific antibodies linking Adamts4 and Dnajb1. All sections were counterstained with DAPI. **(D and E)** Quantification of cells expressing Adamts4 **(D)** and Dnajb1 **(E)** in liver tissues. The data are expressed as mean ± SEM (*P < 0.05, **P < 0.01, I/R versus Sham group).

The expression of seven novel miRNAs identified from bioinformatic analysis was evaluated with RT-qPCR. In younger mice, differential expression of six miRNAs (miR-9-5p, miR-592-5p, miR-124-3p, miR-329-3p, miR-709, and miR-290a-5p) was apparent after hepatic I/R injury (Fig. 4A). In older mice the expression of only three miRNAs (miR-9-5p, miR-329-3p, and miR-290a-5p) was significantly different after hepatic I/R injury (Fig. 4B). In addition, immunofluorescence staining result revealed a significant increase in the expression of two target genes (*Adamts4* and *Dnajb1*) of miR-329-3p after hepatic I/R injury in both young and old mice (Fig. 4C-E). And we also analyzed potential binding sites of miR-329-3p on the two target genes in Supplementary Figure 2.

## Discussion

In this study, we sought to identify differentially expressed miRNA and their plausible target genes after hepatic I/R injury and made a direct comparison between younger and older mice using qPCR and immunofluorescence techniques. The findings of this study provide several avenues for the development of novel therapeutic molecules to minimize the impact of I/R injury, particularly in more vulnerable liver tissues in elderly patients.

The biological mechanisms underlying hepatic I/R injury remain unclear. In recent years, several studies implicated miRNAs in the pathway of injury. Many miRNAs are dysregulated in I/R injury, and may serve as both promising diagnostic biomarkers and potential therapeutic targets 16-18. Here, we identified several miRNAs and target genes using bioinformatic analysis that were differentially regulated following I/R injury. Many of these have not undergone previous investigation in models of liver I/R injury and may present promising targets for future research. Some of the miRNAs we highlighted here have undergone extensive previous exploration. For example, miR-9-5p regulates the expression of *Nfkb3* and plays an important role in the protective effect of sevoflurane in hepatic I/R injury 19. miR-124-3p directly interacts with mmu_circRNA_005186 and *Epha2* and is closely related to hepatic I/R injury and ischemic postconditioning (IPO) 20. The effect of microRNA-592-5p on hippocampal neuron injury following hypoxic-ischemic brain damage in neonatal mice was also recently studied 21. In this study, we explored these miRNAs using bioinformatic tools alone. Future studies including both *in vitro* and *in vivo* experiments are required to validate these early findings.

Many studies have explored the association between older age and increased vulnerability to hepatic liver damage in I/R injury models 22, 23. Here we used three methods to explore the association of age and the severity of injury in a hepatic I/R model in older versus younger mice. This included histopathology, a TUNEL assay, and serum AST/ALT levels. These methods are those widely used in the assessment of hepatic I/R injury 24. We found that some miRNAs related to hepatic I/R injury exhibited differential expression after hepatic I/R injury in both younger and older mice. However, the differential expression of some miRNAs was not consistent with the predicted results of the bioinformatic analysis. This inconsistency in differential expression has been previously reported in I/R injury for both miR-9-5p and miR-124-3p 25, 26. Our data suggested that miR-9-5p, miR-329-3p, and miR-290a-5p may play an important role in hepatic I/R injury in both young and old mice, with the differential expression of miR-329-3p observed most significantly. However, the functional implications of changes in miRNAs expression remained unclear. The predicted target genes (*Adamts4* and *Dnajb1*) of miR-329-3p had a corresponding overexpression in hepatic I/R injury in both younger and older mice. ADAM metallopeptidase with thrombospondin type 1 motif 4 (ADAMTS4) is an inflammation-regulated enzyme, which has previously demonstrated overexpression in the ischemic brain in both human and murine models 27. A recent comprehensive and combined genomic analysis revealed that *DnaJ* heat shock protein family member B1 (*DNAJB1*) is another key gene in the hepatic I/R injury pathway, with likely clinical significance 28. Our data suggest that these pathways may be independent of age-related changes in the severity of I/R injury observed and require further investigation.

In summary, we identified several miRNAs and linked regulatory genes that may be linked to hepatic I/R injury using a bioinformatic analysis. We then explored the differential expression of miR-329-3p and its regulatory genes *Adamts4* and *Dnajb1* after hepatic I/R injury in younger and older mice, where it was upregulated in both groups. miR-329-3p may be a new therapeutic target to mitigate against I/R injury in both younger and older livers. The emergence of miR-329-3p and its regulatory genes therapeutic candidates requires further validation.

## Supplementary Material

Supplementary figures.

## Abbreviations

I/R: ischemia/reperfusion
GO: Gene Ontology
miRNA: MicroRNA
mRNA: messenger RNA
ALT: alanine aminotransferase
AST: aspartate aminotransferase
H&E: hematoxylin and eosin
DNAJB1: DnaJ heat shock protein family member B1
