# Supplementary material for: Decreased miR-329-3p upregulates Adamts4 and Dnajb1 in mouse hepatic I/R injury in an age-independent manner
Source: Int J Med Sci. 2023 Sep 18;20(12):1562–9. doi: 10.7150/ijms.87174 (PMC10583182; doi:10.7150/ijms.87174)
Supplement: Supplementary file 1 — Supplementary figures. [file ijmsv20p1562s1.pdf]

Supplementary Materials

Supplementary Fig. 1. DEMiR-miRTG networks identified from the GSE72315 dataset and miRTarBase. For the miRNA expression profiling dataset, the threshold was determined according to the following values: $P < 0.05$ and fold change ≥ 3.0. A cystoscope was used for visualization.

Supplementary Fig. 2. Potential binding sites of miR-329-3p on *Adamts4* (A-C) and *Dnajb1* (D-F) genes.

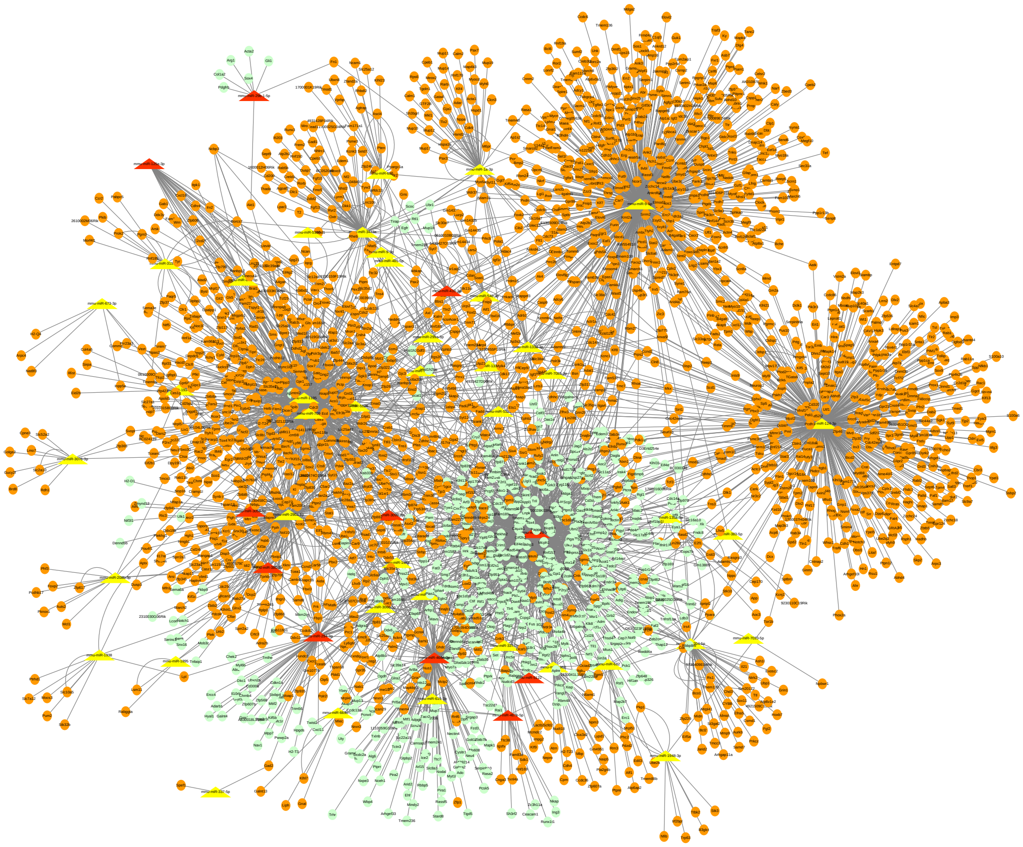

A

```
miRNA  3' uuUUUCCAAUCGACCCACACAa 5'
            |||  | ||:  |||||||
Target 5' acAAATATGAGT--GGTGTGTg 3'
```

B

```
miRNA  3' uuuuuccaaucGACCCACACAa 5'
                     || |||||||
Target 5' gacgccctcttCT-GGTGTGTc 3'
```

C

```
miRNA  3' uuuuuccAAUCGACCCAC-ACAa 5'
                 ||| ||||||| |||
Target 5' catcttaTTACCTGGGTGATGTt 3'
```

D

```
miRNA  3' uuuuuccaaUCGACCCACACaa 5'
                   |||| ||||||
Target 5' tccattggaAGCT-GGTGTGca 3'
```

E

```
miRNA  3' uuUUUCCAAU-CGACCCACACAa 5'
            | |||| |  :|| ||||||
Target 5' gcACAGGTGATTTTGTGTGTGTg 3'
```

F

```
miRNA  3' uuuuuccaaucgaCCCACACaa 5'
                       |||||||
Target 5' agctcctgcccaaGGGTGTGag 3'
```
